# The role of coronary artery calcification score in clinical practice

**DOI:** 10.1186/1471-2261-8-38

**Published:** 2008-12-17

**Authors:** Lieuwe H Piers, Farah Salachova, Riemer HJA Slart, Rozemarijn Vliegenthart, Riksta Dikkers, Frederique AP Hospers, Hjalmar R Bouma, Clark J Zeebregts, Tineke P Willems, Matthijs Oudkerk, Felix Zijlstra, René A Tio

**Affiliations:** 1 Department of Cardiology, University Medical Center Groningen, Groningen, the Netherlands; 2 Department of Nuclear Medicine and Molecular Imaging, University Medical Center Groningen, Groningen, the Netherlands; 3 Department of Radiology, University Medical Center Groningen, Groningen, the Netherlands; 4 Department of Vascular Surgery, University Medical Center Groningen, Groningen, the Netherlands

## Abstract

**Background:**

Coronary artery calcification (CAC) measured by electron-beam computed tomography (EBCT) has been well studied in the prediction of coronary artery disease (CAD). We sought to evaluate the impact of the CAC score in the diagnostic process immediately after its introduction in a large tertiary referral centre.

**Methods:**

598 patients with no history of CAD who underwent EBCT for evaluation of CAD were retrospectively included into the study. Ischemia detection test results (exercise stress test, single photon emission computed tomography or ST segment analysis on 24 hours ECG detection), as well as the results of coronary angiography (CAG) were collected.

**Results:**

The mean age of the patients was 55 ± 11 years (57% male). Patients were divided according to CAC scores; group A < 10, B 10 – 99, C 100 – 399 and D ≥ 400 (304, 135, 89 and 70 patients respectively). Ischemia detection tests were performed in 531 (89%) patients; negative ischemia results were found in 362 patients (183 in group A, 87 in B, 58 in C, 34 in D). Eighty-eight percent of the patients in group D underwent CAG despite negative ischemia test results, against 6% in group A, 16% in group B and 29% in group C. A positive ischemia test was found in 74 patients (25 in group A, 17 in B, 16 in C, 16 in D). In group D 88% (N = 14) of the patients with a positive ischemia test were referred for CAG, whereas 38 – 47% in group A-C.

**Conclusion:**

Our study showed that patients with a high CAC score are more often referred for CAG. The CAC scores can be used as an aid in daily cardiology practice to determine further decision making.

## Background

Since coronary artery disease (CAD) is a major cause of mortality in industrialized western countries, its detection before complications arise has substantial clinical relevance.[1,2] CAD is caused by atherosclerosis and its first manifestations can be found as early as in the second decade of life.[3] Atherosclerotic lesions may progress to such an extent that coronary flow is impaired. The ensuing myocardial ischemia is clinically manifested as chest pain.[4] Chest pain is one of the first symptoms of CAD and also a common complain for which patients seek medical care. Most often an exercise stress test (EST) is the investigation of first choice in the evaluation of chest pain patients. A positive stress test outcome pleads for underlying CAD, whereas a negative test does not necessary rule out obstructive CAD.[5] EST and other non-invasive techniques, such as myocardial perfusion single photon emission computed tomography (SPECT), can only identify patients with advanced CAD.[6,7] Thus, there is a need for a better diagnostic procedure to detect CAD in an earlier stage.

Electron-beam computed tomography (EBCT) can detect and quantify coronary artery calcification (CAC). Several studies found a strong independent association between CAC score and CAD detected with coronary angiography (CAG). [8-10] The sensitivity and specificity of CAC score for detection of CAD in symptomatic patients appeared to be 93% and 88%, respectively.[9] The negative predictive value of EBCT varies from 95% to 100%. [10,11]

Next to being of diagnostic value, CAC score is also an important prognostic determinant for coronary events. The measurement of CAC is an appropriate initial screening tool in patients at increased coronary risk.[12] Symptomatic patients with excessive CAC score carry a high risk for future events.[13] A negative CAC score has been associated with a very low event rate of 0.4 events per 1000 per year.[14] The negative predictive value of CAC score is of great value for cardiologists as well as general practitioners, which means that CAC score is an important identifier of low risk patients.

As yet, there is little information about the implementation of EBCT for clinical decision making in routine clinical practice. The question whether negative or positive EBCT outcome influences the decision of the clinician for further diagnostic investigations has still to be answered. The aim of this study is to evaluate the contribution of EBCT derived CAC score in the management of patients suspected of CAD by clinicians.

## Methods

**Figure 1:**
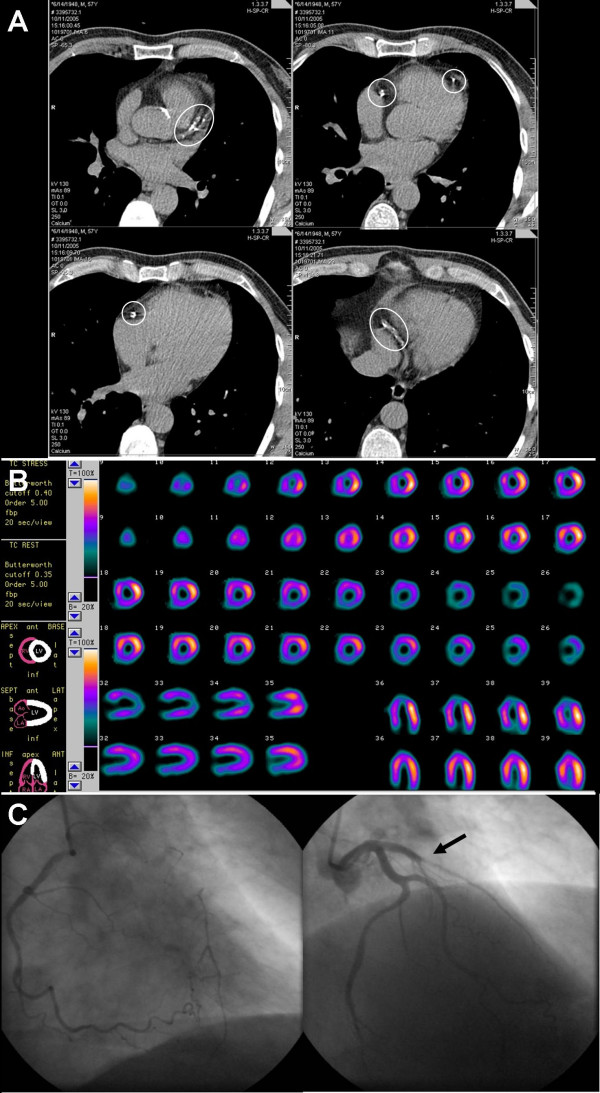
**Images of a 66-year-old symptomatic man who had a coronary artery calcification score of 671; A: electron-beam computed tomography, B: myocardial perfusion single photon emission computed tomography (SPECT), C: coronary angiography (CAG)**. Circles define regions of coronary calcification. SPECT demonstrated myocardial perfusion defect in the apex and anteroseptal region after bicycle stress test (upper rows). These were completely reversible during myocardial perfusion rest test. CAG revealed 1-vessel disease of the left anterior descending artery, with collateral filling from the right coronary artery. Arrow indicates the occlusion of the left anterior descending artery. Based on these results, the patient was accepted for coronary artery bypass graft surgery.

Between November 2004 and March 2006, 1008 patients were referred to the University Medical Centre Groningen (a tertiary referral centre) for cardiac EBCT. Thirty-six subjects with a history of CAD were excluded (history of myocardial infarction, percutaneous coronary intervention or coronary artery bypass graft). In addition patients referred for evaluation of aortic valve stenosis (n = 67), or atrial dimensions (n = 55) were excluded, as well as those being referred for pre-operative screening (n = 70) or research purposes (n = 182). The study population of our retrospective observational study consisted of 598 patients referred for the evaluation of CAD. They underwent additional diagnostic procedures for evaluation of myocardial ischemia, as being clinically relevant according to the treating cardiologist; e.g. EST, ST segment analysis on 24 hours ECG registration, and myocardial perfusion SPECT. If judged clinically relevant, a CAG was performed. The Framingham Risk Score was calculated for each patient to determine the 10-year cardiovascular risk.[15] Figure 1 shows images of performed diagnostic procedures from one of the included patients. Due to the nature of this study (retrospective observational study), the institutional review board concluded that the study was exempt from the obtainment of ethical approval.

### Myocardial ischemia tests

#### Exercise stress test

Patients underwent EST in accordance with the guidelines of the American Heart Association.[16] ECG was recorded at rest and during exercise. Exercise began at 25 watt, followed by an increase of 10 to 25 watt every 2 minutes depending on patient's age, gender, and weight. ST segments were monitored continuously. The exercise was terminated upon maximum exertion (e.g. exhaustion, dyspnoea), in the absence of ECG changes suggestive for ischemia or other mandatory end-points (e.g. drop in systolic blood pressure (≥ 10 mm Hg), anginal complaints, ischemic ST segment changes, serious arrhythmias, urgent request to stop). In case of a maximum heart rate < 85% of the predicted maximum or non-diagnostic ECG changes, the test was considered to be non-conclusive.

#### ST segment analysis on twenty four hour ECG registration

Twenty-four hour ECG registration was performed using 4 channel Holter recorders (GE Marquette series 8500, Milwaukee, WI). Modified leads aVF, V1 and V5 were used. The ECG was analysed on GE Marquette MARS analyser and reviewed by an experienced analyst and supervised by a cardiologist. The rule of 1-1-1 was applied as a criterium for ST-segment analysis.[17]

#### Myocardial perfusion single photon emission computed tomography

A standardized protocol was used for myocardial perfusion SPECT. A rest SPECT was performed, after injection of 600 MBq of ^99 m^Tc-tetrofosmin, including gated images in 8 frames at an R-R interval of ± 10%. The next day stress SPECT was performed after adenosine or bicycle exercise. SPECT images were acquired one hour after tracer administration using a double headed gamma-camera (Ecam, Siemens Medical Systems, Chicago, IL) equipped with low-energy high-resolution collimators. The camera heads were in perpendicular position. Other acquisition parameters were: 32 steps rotation, 20 sec per step, 128 × 128 matrix size, rotation from the 45° right anterior oblique to the 135° left posterior oblique position with the patient laying supine. SPECT images were reconstructed after filtered-back-projection using a Butterworth 0.30/6 filter. All data were re-orientated in order to produce short-axis, horizontal long-axis and vertical long-axis sections. The myocardial perfusion SPECT images were analysed by a panel of nuclear medicine physicians and cardiologists.

The quantitative gated SPECT analysis program was used for assessing myocardial perfusion.[18] Segmental myocardial perfusion was analyzed quantitatively and segmental tracer activity was categorized on a 4-point scale of 1 = normal tracer activity > 75%, 2 = 50% to 75% tracer activity, 3 = 25% to 50% tracer activity, and 4 = tracer activity < 25%. Perfusion defects on stress images were considered present when tracer activity was < 75% of maximum. When significant fill-in (>10%) of perfusion defects was observed on the images at rest, segments were classified as ischemic, whereas defects without fill-in (≤ 10%) were considered scar tissue.

The results of EST, ST segment analysis on 24 hours ECG registration or myocardial perfusion SPECT were combined and divided into 3 groups: (1) negative, (2) non-conclusive or (3) positive for myocardial ischemia. A non-conclusive test overruled another negative test, except for a negative SPECT. A positive test overruled a negative or non-conclusive test in any case.

### Electron-beam computed tomography

All patients underwent EBCT scan (C-150 Imatron, General Electrics, San Francisco, CA). Beginning from the aortic root, 38 images were obtained with 100 ms scan time and 3 mm slice thickness. During a single breath-hold, the images were acquired at 80% of the R-R interval, using ECG-triggering. Scans were made with 130 kV and 895 mAs. The entire procedure lasted 15 minutes per patient, including the time for assessing CAC score. A CAC score was determined according to the methods described by Agatston et al, using a commercially available calcium scoring software package (SmartScore, GE Healthcare, San Francisco, CA).[19] CAC scores were divided into < 10, 10–99, 100–399 and ≥ 400. In case of a CAC score of ≥ 400 a CAG was advised.

### Coronary angiography

CAG was performed according to standard procedure. At least two orthogonal views of the right coronary artery and at least five views of the left coronary system were made. A significant stenosis was defined as a luminal narrowing of ≥ 50%. In case of such significant stenosis, the patients were considered to have significant CAD. In case no CAG was performed, presence of CAD was determined to be unknown.

### Statistical analysis

We analysed data with SPSS version 12.01. Data are reported as mean ± SD for parametric variables, as median and interquartile range for non-parametric variables, and in the case of nominal variables as percent frequency. In case of nominal variable, we used the chi-square test for analysis of differences between 2 groups. To compare not normally distributed data between 2 groups, the Mann-Whitney U test was used and a Students t-test in case of normally distributed data. To analyze differences between > 2 groups, we used the Kruskall-Wallis test in case of skewed or dichotomous data, and ANOVA in case of normally distributed data. A p-value < 0.05 was considered to be significant. The Framingham Risk Score was used to calculate the 10-year risk of coronary heart disease.[15] Multivariable Cox regression was used to evaluate the association between clinical variables and the probability of undergoing CAG. CAC score group, ischemia test result and traditional cardiovascular risk factors (age, sex, diabetes mellitus, hypertension, hypercholesterolemia, smoking habit and family history of cardiovascular disease) were added to the model. In another model CAC score groups were changed for continuous CAC score. Data are expressed as hazard ratios (HR) with 95% confidence intervals (CI). A p value < 0.05 was considered statistically.

## Results

**Figure 2:**
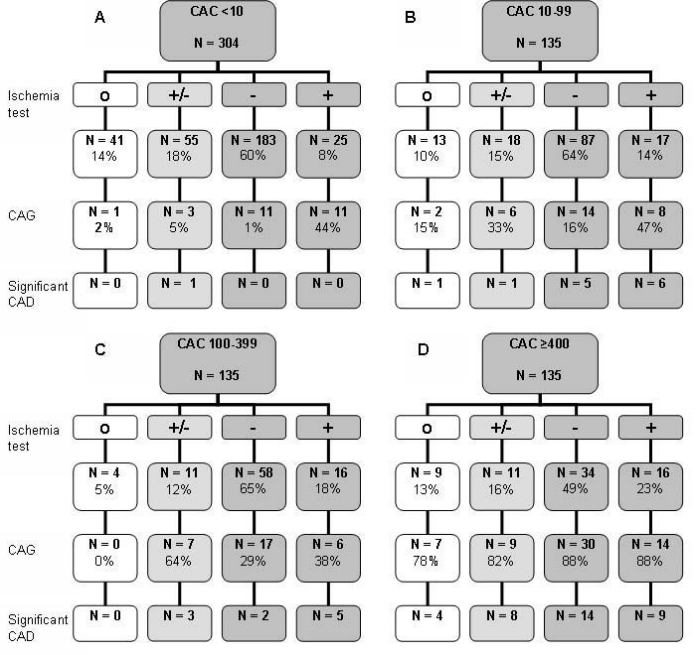
5**98 patients, divided into 4 groups (A – D) underwent electron-beam computed tomography**. In 531 an ischemia test was performed, being either non-conclusive, negative or positive. One-hundred-forty-six patients underwent CAG and in 59 significant CAD was found. CAC = coronary artery calcification; CAD = coronary artery disease; CAG = coronary angiography; O = no ischemia test; +/- = non-conclusive result; **- **= negative ischemia test; **+ **= positive ischemia test.

In total, 598 patients met the inclusion criteria (age 55 ± 11 years, 57% male). Clinical characteristics of the patients are shown in table 1. Patients were divided into four groups based on their CAC score: group A CAC score < 10; group B CAC score 10–99; 100–399; group D CAC scores ≥ 400 (304, 135, 89 and 70 patients respectively). In 531 (89%) patients additional diagnostic tests were performed to screen for myocardial ischemia; 446 EST, 135 ST segment analyses on 24 hours ECG registration, and 135 myocardial perfusion SPECT. One-hundred-forty-six patients (24%) underwent CAG. Figure 2 shows the results of ischemia tests divided per CAC score group (A-D).

**Table 1:** Baseline patient characteristics of whole study population and divided into group A (CAC score < 10), group B (CAC score 10–99), group C (CAC score 100–399), group D (CAC score ≥ 400)

	***All***	**A**	**B**	**C**	**D**
**N**	***598***	**304**	**135**	**89**	**70**
Male*	***338 (57%)***	156 (51%)	80 (59%)	60 (67%)	42 (60%)
Age (years)*	***55 ± 11***	50 ± 11	56 ± 10	62 ± 10	63 ± 10
CAC score*	***13 (0–158)***	0 (0–1)	44 (24–65)	224 (148–295)	693 (511–1284)
Risk factors					
Hypertension*	***231 (57%)***	138 (45%)	74 (55%)	60 (67%)	55 (79%)
Diabetes	***47 (12%)***	24 (8%)	20 (15%)	11 (12%)	8 (11%)
Hypercholesterolemia*	***164 (41%)***	100 (33%)	61 (45%)	40 (45%)	41 (59%)
Smoking	***117 (29%)***	109 (36%)	51 (38%)	39 (44%)	25 (36%)
Family history of CVD	***209 (52%)***	159 (52%)	67 (50%)	39 (44%)	42 (60%)
10-year risk of CHD (%)*	***6 (3–10)***	4 (2–8)	6 (4–10)	10 (6–12)	8 (5–12)
CVD history					
Atrial fibrillation	***25 (7%)***	12 (4%)	2 (2%)	5 (6%)	6 (9%)
Cerebrovascular disease	***16 (4%)***	13 (4%)	9 (7%)	6 (7%)	2 (3%)
Other	***36 (10%)***	17 (6%)	12 (9%)	10 (11%)	9 (13%)
Ischemia test	***531 (89%)***	263 (87%)	122 (90%)	85 (96%)	61 (87%)
Negative	***362 (61%)***	183 (60%)	87 (64%)	58 (65%)	34 (49%)
Non-conclusive	***95 (16%)***	55 (18%)	18 (13%)	11 (12%)	11 (16%)
Positive*	***74 (12%)***	25 (8%)	17 (13%)	16 (18%)	16 (23%)
CAG*	***146 (24%)***	26 (9%)	30 (22%)	30 (34%)	60 (86%)

Values are mean ± SD or median (interquartile range). CAC = coronary artery calcification, CAG = coronary angiography, CHD = coronary heart disease, CVD = cardiovascular disease. * Significant difference between groups, p < 0.05. Framingham Risk Score was used to determine 10-year risk of CHD. Other: cardiovascular history includes valvular diseases, arrhythmias, peripheral arterial disease, dilated cardiomyopathy, heart failure, myocarditis, pericarditis, and pulmonary embolism.

No cardiovascular risk factors were present in 72 (12%) patients. At least one cardiovascular risk factor was present in 526 (88%) patients: 150 (25%) had one and 376 (63%) had two or more risk factors. Typical chest pain was recorded in 127 (21%) patients, atypical chest pain in 388 (65%) and 83 (14%) patients had no chest pain. This last group was referred for cardiac EBCT based on high cardiovascular risk profile, and/or abnormal ECG.

### Coronary artery calcification and coronary angiography

**Figure 3:**
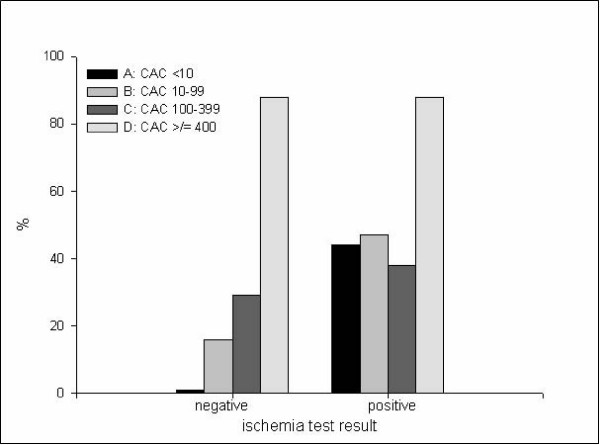
**Percentage of patients of group A (CAC score < 10), group B (CAC score 10–99), group C (CAC score 100–399), group D (CAC score ≥ 400) undergoing coronary angiography in case the ischemia test result was negative or positive**. CAC = coronary artery calcification.

The probability to undergo CAG increases if a patient belongs to a higher CAC score group (p < 0.001, table 2); 9% of group A underwent CAG, 22% of group B, 34% of group C and 86% of group D. If we consider the patients with a negative ischemia test result, the probability of undergoing a CAG also increases with CAC score (p < 0.001, figure 3); 1% of group A underwent CAG, 16% of group B, 29% of group C and 88% of group D. The same applies to the patients with a positive ischemia test (p < 0.05); 44% of group A underwent CAG, 47% of group B, 38% of group C and 88% of group D.

**Table 2:** CAC score and results of ischemia tests in group A (CAC score < 10), group B (CAC score 10–99), group C (CAC score 100–399) and group D (CAC score ≥ 400) according to CAG referral

	**CAG referral**					
		***Yes***			***No***	
	***N***		***CAC score***	***N***		***CAC score***
Total population*	146		269 (37 – 629)	452		1 (0 – 48)
Group A†	26		0 (0 – 2)	278		0 (0 – 1)
Group B	30		47 (28 – 73)	105		44 (23 – 64)
Group C†	30		256 (161 – 354)	59		203 (140 – 268)
Group D†	60		771 (538 – 1398)	10		568 (456 – 729)
						
No ischemia test*	10		642 (70 – 1198)	57		0 (0 – 20)
Ischemia test*	136		256 (31 – 613)	395		1 (0 – 53)
Negative*	72		315 (58 – 595)	290		1 (0 – 51)
Non-conclusive*	25		258 (32 – 831)	70		0 (0 – 11)
Positive†	39		130 (7 – 620)	35		57 (0 – 192)

Values are median (interquartile range). CAC = coronary artery calcification, CAG = coronary angiography. Significant difference in CAC score; * p < 0.001, † p < 0.05

Twenty-five patients in group A had a positive ischemia test. Eleven of these patients were referred for CAG, their CAC score was not significantly different from those not referred for CAG; 1 (0 – 3) versus 0 (0 – 3). In group A 183 patients had a negative ischemia test; 11 of which underwent CAG. The CAC score did not differ between those referred or not; 0 (0 – 2) and 0 (0 – 1) respectively. Moreover, this applies also to group B, C and D.

If we consider all patients with a CAC score ≥ 10, 49 patients had a positive ischemia test. Twenty-eight of them were referred for CAG, they had a significant higher CAC score as the 21 that were not referred; 428 (83 – 892) versus 121 (69 – 222) (p < 0.05). Interestingly, the 61 patients with a CAC score ≥ 10, who were referred for CAG despite a negative ischemia test had a CAC of 389 (119 – 639) versus 66 (40 – 173) in the 118 patients that were not referred for CAG (p < 0.001). The same applies to the whole population; the 72 patients who were referred for CAG despite a negative ischemia test had a CAC score of 315 (58 – 595) against 1 (0 – 51) in the 290 patients that were not referred for CAG (p < 0.001). If the ischemia test was non-conclusive patients referred for CAG had higher CAC scores than those not; 258 (32 – 831) and 0 (0 – 11) respectively (p < 0.001).

In 67 out of 598 patients, no ischemia detection tests were performed. Ten of them underwent CAG (CAC score 641 (70 – 1198)) and significant CAD was found in 5 of them (CAC score 1016 (407 – 2633)).

### Multivariable Cox regression

Regression analysis showed a significant association between CAC score group (A-D) and CAG referral (p < 0.001), see table 3. If ischemia test results were added to the model, the association did not change. Ischemia test results were also significantly associated to CAG referral (p < 0.001). Also, if traditional cardiovascular risk factors were taken into account, the association between CAC score groups and CAG referral did not change. Because a CAG was advised in case of a CAC score of ≥ 400, we performed regression analyses for the population with a CAC score < 400. The association between CAC score group (A-C) and CAG referral remained significant after correction for ischemia test results and traditional cardiovascular risk factors (p < 0.05), see table 3.

**Table 3:** Hazard ratios: Cox regression

		**All CAC score groups included**			**CAC score group A-C included**		
	
***Univariable***		***HR***	***(95% CI)***	***P-value***	***HR***	***(95% CI)***	***P-value***
CAC score group	A	reference			reference		
	B	3.06	(1.73–5.41)	<0.001	3.06	(1.73–5.41)	<0.001
	C	5.44	(3.00–9.86)	<0.001	5.44	(3.00–9.86)	<0.001
	D	64.2	(29.4–140)	<0.001	-		
							
***Multivariable***		*HR*	*(95% CI)*	*P-value*	*HR*	*(95% CI)*	*P-value*
CAC score group	A	reference			reference		
	B	3.28	(1.19–4.37)	<0.001	2.38	(1.23–4.60)	0.01
	C	3.88	(1.86–8.11)	<0.001	4.16	(1.96–8.84)	<0.001
	D	51.4	(19.9–133)	<0.001	-		
Ischemia test result	Negative	reference			reference		
	Non-conclusive	1.84	(0.96–3.54)	<0.001	2.12	(1.07–4.19)	0.03
	Positive	4.38	(2.30–8.35)	<0.001	5.21	(2.65–10.3)	<0.001
Age		1.00	(0.98–1.04)	0.64	1.00	(0.97–1.03)	0.94
Male		1.30	(0.74–2.26)	0.36	1.26	(0.70–2.29)	0.44
Hypertension		1.09	(0.62–1.90)	0.77	1.20	(0.67–2.16)	0.55
Diabetes		2.13	(1.02–4.45)	<0.05	1.80	(0.82–3.98)	0.14
Hypercholesterolemia		1.02	(0.59–1.75)	0.96	1.11	(0.62–1.96)	0.73
Smoking		1.44	(0.83–2.50)	0.20	1.50	(0.83–2.68)	0.18
Family history of CVD		1.03	(0.61–1.74)	0.91	0.98	(0.56–1.70)	0.94

CAC = coronary artery calcification, CVD = cardiovascular disease. Group A: CAC score < 10; group B: CAC score 10–99; group C: CAC score 100–399; group D: CAC score ≥ 400.

Regression analysis showed a significant association between continuous CAC score and the probability of undergoing CAG (HR 1.01; 95% CI 1.00 – 1.01, p < 0.001). Adjustment for ischemia test result and traditional risk factors did not change the association between CAC score and probability of undergoing CAG (HR 1.01; 95% CI 1.00 – 1.01, p < 0.001). These results did not change if we only included patients with a CAC score < 400 into the regression analysis.

## Discussion

We found an important impact of CAC measurements on clinical decision making, whether to perform additional invasive CAG or not. Especially in patients with a negative ischemia test result CAG referral was more likely chosen in case of a higher CAC score. Ischemia test results do not influence the clinical decision making in case a patient has a CAC score ≥ 400.

The value of CAC for the prediction of CAD and cardiovascular events has been well-established. CAC identifies anatomical correlates of CAD. Moreover, it has been shown that the CAC score is an important predictor of ischemia on SPECT.[20] However, there is no significant difference in the overall diagnostic performance between EBCT and SPECT.[9]

There are several benefits of EBCT over cardiac stress tests. First, coronary blood flow does not have to be compromised for the detection. Second, the accuracy of EST is limited by the ability of the patient to exercise or to baseline ECG abnormalities, whereas EBCT does not have these limitations. Third, other important possible sources of the chest pain, such as pneumonia, pericarditis, and aortic disease, can be seen on the EBCT.

The focus of our study was to obtain information about the impact of EBCT on the clinical decision for further diagnostic investigations. We found that the CAC score is an important determinant for the decision whether to perform CAG, as is shown in figure 2, especially in case of a negative stress test. In addition, in the total population the CAC score was higher in patients undergoing CAG, independent of ischemia test results. Furthermore, 26 patients underwent CAG from group A, despite a negative EBCT result, and significant CAD was found in only 1 of them. None of the 183 patients in group A with a negative ischemia test (11 CAG were performed, 6%), appeared to have significant CAD, in contrast to 21 of 179 patients with a CAC score ≥ 10 (61 CAG were performed, 34%). In addition, none of the patients in group A with a positive ischemia test result appeared to have significant CAD.

Due to its high sensitivity, EBCT might help to identify false positive ischemia test results.[10,11] However, the specificity remains low; in our study 35 of 135 (26%) patients with a CAC score ≥ 400 showed significant CAD on CAG. Moreover, previous reports have shown that 31–47% patients with a CAC score ≥ 400 had a normal SPECT result.[20,21] As a marker of CAD, the CAC score can predict long-term coronary risk. However, the rather low specificity does not make it a good predictor of obstructive CAD. Sending patients with abnormal EBCT results to CAG without having positive ischemia test results is not beneficial, as the patient will be exposed to the risk of an invasive procedure without the need for revascularization.[22] Moreover, 1 patient had significant CAD, despite a CAC score of 0. This underlines the fact that clinical presentation and cardiovascular risk factors have always to be taken into account. This finding is confirmed by another previous study, where CAD was diagnosed in 9 patients (age 50 ± 9, 77% female) with a CAC score of 0. [23] Conversely, our patient was 71-year old women with a CAC score of 0. This illustrates that CAD can be diagnosed independent of low scores of coronary calcification, although it is very unlikely.

The potential influence of CAC score on decision making in stable patients without ischemia remains to be elucidated. The negative predictive value of SPECT has been well established. [24] On the other hand, the prognostic value of increased CAC scores has also been extensively shown. [11] The question 'What to do in case of a high CAC score and a negative SPECT' can not easily been answered. In several studies the diagnostic values of SPECT and CAC score have been compared. [20,25,26] Specificity and sensitivity of CAC score seems higher or at least equivalent in most studies. In addition, the latter study suggests that CAC score may be useful preceding SPECT, in order to filter out the patients with a low probability at a positive SPECT. However, it is not known whether patients with a high CAC score benefit from revascularization; it is known that patients with positive ischemia test results benefit from revascularization. [24] So, the specific role and place of the CAC score in the diagnostic process has to be determined.

### Study limitations

The retrospective design of this study may have introduced bias in patient referral for CAG. Indeed, the decision to perform CAG was not only based on CAC score, but also clinical judgement, cardiovascular risk, and symptomatic status. Due to the nature of this study this can not be prevented. Only 70 patients with a CAC score of ≥ 400 were present. The clinical advice to perform a CAG in case of a CAC ≥ 400 has introduced a bias. However, despite this clinical advice, only 60 of these 70 patients were referred for CAG. In comparison, 14 patients of 221 with CAC score of 0 were referred for CAG. Furthermore, not all patients underwent ischemia tests. However, these data give a good representation of the impact of EBCT on daily clinical decision making.

## Conclusion

Our data suggest that CAC scores can be used in daily clinical practice, in combination with other available diagnostic information, as an aid in further decision making. Future studies to develop the CAC score as a means to triage patients towards more expensive and complex diagnostic tools are needed and will determine the ultimate role of the CAC score in cardiovascular medicine.

## Abbreviations

CAC: coronary artery calcification; CAD: coronary artery disease; CAG: coronary angiography; EBCT: electron-beam computed tomography; EST: exercise stress test; SPECT: single photon emission computed tomography.

## Competing interests

The authors declare that they have no competing interests.

## Authors' contributions

LP and FS drafted the manuscript. RT and FZ participated in the conception and design of the study. FS, LP, RV, FH and HB performed the collection, analysis and interpretation of data. CZ, RS, RV, RD, TW, MO, FZ, RT helped to draft the manuscript and added important intellectual content. All authors read and approved the final manuscript.

## Pre-publication history

The pre-publication history for this paper can be accessed here:



## References

[B1] Froelicher VF, Thomas MM, Pillow C, Lancaster MC (1974). Epidemiologic study of asymptomatic men screened by maximal treadmill testing for latent coronary artery disease. Am J Cardiol.

[B2] McHenry PL, O'Donnell J, Morris SN, Jordan JJ (1984). The abnormal exercise electrocardiogram in apparently healthy men: a predictor of angina pectoris as an initial coronary event during long-term follow-up. Circulation.

[B3] Wexler L, Brundage B, Crouse J, Detrano R, Fuster V, Maddahi J, Rumberger J, Stanford W, White R, Taubert K (1996). Coronary artery calcification: pathophysiology, epidemiology, imaging methods, and clinical implications. A statement for health professionals from the American Heart Association. Writing Group. Circulation.

[B4] Ross R (1993). The pathogenesis of atherosclerosis: a perspective for the 1990s. Nature.

[B5] Gibbons RJ, Balady GJ, Beasley JW, Bricker JT, Duvernoy WF, Froelicher VF, Mark DB, Marwick TH, McCallister BD, Thompson PD, Winters WL, Yanowitz FG, Ritchie JL, Gibbons RJ, Cheitlin MD, Eagle KA, Gardner TJ, Garson A, Lewis RP, O'Rourke RA, Ryan TJ (1997). ACC/AHA Guidelines for Exercise Testing. A report of the American College of Cardiology/American Heart Association Task Force on Practice Guidelines (Committee on Exercise Testing). J Am Coll Cardiol.

[B6] Mahmarian JJ, Boyce TM, Goldberg RK, Cocanougher MK, Roberts R, Verani MS (1990). Quantitative exercise thallium-201 single photon emission computed tomography for the enhanced diagnosis of ischemic heart disease. J Am Coll Cardiol.

[B7] Weiner DA, Ryan TJ, McCabe CH, Luk S, Chaitman BR, Sheffield LT, Tristani F, Fisher LD (1987). Significance of silent myocardial ischemia during exercise testing in patients with coronary artery disease. Am J Cardiol.

[B8] Guerci AD, Spadaro LA, Goodman KJ, Lledo-Perez A, Newstein D, Lerner G, Arad Y (1998). Comparison of electron beam computed tomography scanning and conventional risk factor assessment for the prediction of angiographic coronary artery disease. J Am Coll Cardiol.

[B9] Heijenbrok-Kal MH, Fleischmann KE, Hunink MG (2007). Stress echocardiography, stress single-photon-emission computed tomography and electron beam computed tomography for the assessment of coronary artery disease: a meta-analysis of diagnostic performance. Am Heart J.

[B10] Rumberger JA, Sheedy PF, Breen JF, Schwartz RS (1995). Coronary calcium, as determined by electron beam computed tomography, and coronary disease on arteriogram. Effect of patient's sex on diagnosis. Circulation.

[B11] Haberl R, Becker A, Leber A, Knez A, Becker C, Lang C, Bruning R, Reiser M, Steinbeck G (2001). Correlation of coronary calcification and angiographically documented stenoses in patients with suspected coronary artery disease: results of 1,764 patients. J Am Coll Cardiol.

[B12] Geluk CA, Dikkers R, Kors JA, Tio RA, Slart RH, Vliegenthart R, Hillege HL, Willems TP, de Jong PE, van Gilst WH, Oudkerk M, Zijlstra F (2007). Measurement of coronary calcium scores or exercise testing as initial screening tool in asymptomatic subjects with ST-T changes on the resting ECG: an evaluation study. BMC Cardiovasc Disord.

[B13] Mohlenkamp S, Lehmann N, Schmermund A, Pump H, Moebus S, Baumgart D, Seibel R, Gronemeyer DH, Jockel KH, Eberl R (2003). Prognostic value of extensive coronary calcium quantities in symptomatic males – a 5-year follow-up study. Eur Heart J.

[B14] Church TS, Levine BD, McGuire DK, Lamonte MJ, Fitzgerald SJ, Cheng YJ, Kimball TE, Blair SN, Gibbons LW, Nichaman MZ (2007). Coronary artery calcium score, risk factors, and incident coronary heart disease events. Atherosclerosis.

[B15] (2001). Executive Summary of The Third Report of The National Cholesterol Education Program (NCEP) Expert Panel on Detection, Evaluation, And Treatment of High Blood Cholesterol In Adults (Adult Treatment Panel III). JAMA.

[B16] Fletcher GF, Balady G, Froelicher VF, Hartley LH, Haskell WL, Pollock ML (1995). Exercise standards. A statement for healthcare professionals from the American Heart Association. Writing Group. Circulation.

[B17] Cohn PF (1987). Total ischemic burden: pathophysiology and prognosis. Am J Cardiol.

[B18] Germano G, Kiat H, Kavanagh PB, Moriel M, Mazzanti M, Su HT, Van Train KF, Berman DS (1995). Automatic quantification of ejection fraction from gated myocardial perfusion SPECT. J Nucl Med.

[B19] Agatston AS, Janowitz WR, Hildner FJ, Zusmer NR, Viamonte M, Detrano R (1990). Quantification of coronary artery calcium using ultrafast computed tomography. J Am Coll Cardiol.

[B20] He ZX, Hedrick TD, Pratt CM, Verani MS, Aquino V, Roberts R, Mahmarian JJ (2000). Severity of coronary artery calcification by electron beam computed tomography predicts silent myocardial ischemia. Circulation.

[B21] Geluk CA, Dikkers R, Perik PJ, Tio RA, Gotte MJ, Hillege HL, Vliegenthart R, Houwers JB, Willems TP, Oudkerk M, Zijlstra F (2008). Measurement of coronary calcium scores by electron beam computed tomography or exercise testing as initial diagnostic tool in low-risk patients with suspected coronary artery disease. Eur Radiol.

[B22] Fox K, Garcia MA, Ardissino D, Buszman P, Camici PG, Crea F, Daly C, de Backer G, Hjemdahl P, Lopez-Sendon J, Marco J, Morais J, Pepper J, Sechtem U, Simoons M, Thygesen K, Priori SG, Blanc JJ, Budaj A, Camm J, Dean V, Deckers J, Dickstein K, Lekakis J, McGregor K, Metra M, Morais J, Osterspey A, Tamargo J, Zamorano JL (2006). Guidelines on the management of stable angina pectoris: executive summary: the Task Force on the Management of Stable Angina Pectoris of the European Society of Cardiology. Eur Heart J.

[B23] Rubinshtein R, Gaspar T, Halon DA, Goldstein J, Peled N, Lewis BS (2007). Prevalence and extent of obstructive coronary artery disease in patients with zero or low calcium score undergoing 64-slice cardiac multidetector computed tomography for evaluation of a chest pain syndrome. Am J Cardiol.

[B24] Metz LD, Beattie M, Hom R, Redberg RF, Grady D, Fleischmann KE (2007). The prognostic value of normal exercise myocardial perfusion imaging and exercise echocardiography: a meta-analysis. J Am Coll Cardiol.

[B25] Shavelle DM, Budoff MJ, LaMont DH, Shavelle RM, Kennedy JM, Brundage BH (2000). Exercise testing and electron beam computed tomography in the evaluation of coronary artery disease. J Am Coll Cardiol.

[B26] Berman DS, Wong ND, Gransar H, Miranda-Peats R, Dahlbeck J, Hayes SW, Friedman JD, Kang X, Polk D, Hachamovitch R, Shaw L, Rozanski A (2004). Relationship between stress-induced myocardial ischemia and atherosclerosis measured by coronary calcium tomography. J Am Coll Cardiol.

